# Delayed pneumocephalus induced by repeated percutaneous aspiration after spinal surgery: A case report and literature review

**DOI:** 10.1097/MD.0000000000026322

**Published:** 2021-06-11

**Authors:** Yu Zhao, Liming Cao, Qi Sheng, Ruifang Liu, Gaolei Dong, Xibao Tong

**Affiliations:** a Department of Neurology, Shenzhen Third People's Hospital; b Department of Neurology, The Second Affiliated Hospital, Southern University of Science and Technology; c Department of Neurology, The First Affiliated Hospital of Shenzhen University; d Department of Neurology, The Third Affiliated Hospital of Shenzhen University, Shenzhen; e Department of Internal Medicine, People's Hospital of Xilin County, Baise, China.

**Keywords:** case report, delayed pneumocephalus, percutaneous aspiration, severe headache, spine surgery

## Abstract

**Rationale:**

Severe tension pneumocephalus can lead to drowsiness, coma, and even brain hernia and death. The occurrence of delayed pneumocephalus after spinal surgery is rarely reported and often ignored. Herein, we report a case of delayed pneumocephalus after repeated percutaneous aspiration following spinal surgery.

**Patient concerns:**

A 55-year-old man was admitted in October 2020 because of aggravation in bilateral lower limb weakness and dysuria for seven days. He was diagnosed with liver cancer a year ago, and he underwent several operations because of tumor recurrence. The patient underwent thoracic vertebrae tumor excision on this admission, and no cerebrospinal fluid leakage was discovered during surgery. After the third drainage by percutaneous aspiration, the patient complained of severe headache and vomiting on postoperative day 16.

**Diagnosis:**

Emergency brain computed tomography revealed massive pneumocephalus.

**Interventions:**

Thereafter, suction drainage was discontinued, and he was placed on bed rest and administered intravenous mannitol.

**Outcomes:**

Repeated computed tomography showed complete resolution of the pneumocephalus after five days.

**Lessons:**

Wound exudates and cystic fluid after spinal surgery should be differentiated from cerebrospinal fluid leakage. Reckless percutaneous aspirations can form pneumocephalus in patients with an occult dural injury, and pneumocephalus can occur up to 16 days after surgery. Early diagnosis of pneumocephalus is crucial to avoid severe consequences.

## 1 Introduction

Pneumocephalus is defined as the presence of gas within any of the transcranial compartments, including the intraventricular, intraparenchymal, subarachnoid, and subdural spaces. Severe tension pneumocephalus can lead to drowsiness, coma, brain hernia, and death.^[1,2]^ Dural injury is an important etiological factor for pneumocephalus. To date, delayed pneumocephalus secondary to drainage with percutaneous aspiration has not been reported. There have been recent advances in the instrumentation for spinal surgery, and increasing number of spinal surgeries have been performed. Consequently, surgeons face increasing numbers of dural tears and cerebrospinal fluid (CSF) leaks; CSF leakage secondary to a dural injury can cause pneumocephalus.^[3,4]^ Most pneumocephalus occur within 1 to 3 days^[4–7]^ after spinal surgery, and cases complicated by delayed pneumocephalus are rarely reported. However, there is a paucity of data on how to avoid the pneumocranium after spinal surgery. Here we present a case of delayed pneumocephalus after repeated percutaneous aspiration following spinal surgery. Furthermore, we present a literature review regarding this to summarize our experiences and lessons.

## 2 Methods

The patient signed the informed consent. The study design was approved by the ethics review board of Shenzhen Third People's Hospital (No: 2020-248).

## 3 Case presentation

A 55-year-old man was admitted in October 2020 because of aggravation in bilateral lower limb weakness and dysuria for seven days. He had a history of kidney stones, multiple calculi of the bile duct, hepatolithiasis, lipomatosis, chronic viral hepatitis B, and liver cirrhosis. He had no history of hereditary diseases or drug abuse. He was diagnosed with liver cancer in September 2019 and underwent surgery. Postoperative pathology showed moderately differentiated hepatocellular carcinoma. Owing to the relapse of liver cancer, the patient underwent a transcatheter arterial chemoembolization three months later. The patient underwent a local excision and pedicle screw fixation for thoracic vertebral metastases in July 2020, and he underwent intravascular embolization for liver cancer three months later.

**Figure 1:**
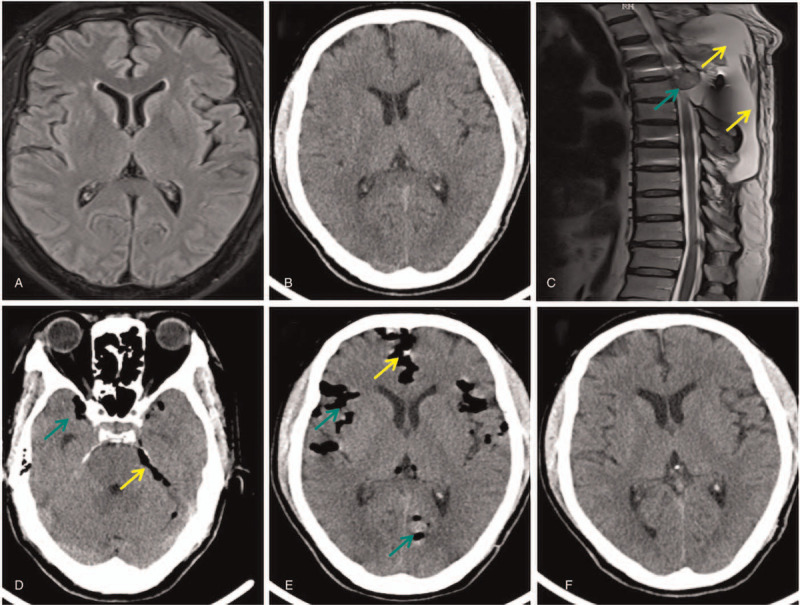
Neuroimaging results before and after the occurrence of pneumocephalus. Preoperative brain magnetic resonance imaging (MRI, A) and computed tomography (CT, B) showing no obvious abnormality. MRI of thoracic vertebra (C) showing roundish nodules (green arrow) in the right foramen intervertebral region of T4–5, which are recurrent or residual tumors, and subcutaneous hydrops (yellow arrows) in the dorsal side of T3–8 vertebrae. Brain CT 16 days after surgery showing massive air densities in the lateral fissure (D and E, green arrows), circle cistern (D, yellow arrow), and anterior longitudinal fissure (E, yellow arrows). Repeated head CT 5 days after pneumocephalus showing complete resolution of the pneumocephalus (F).

On admission, physical examination showed a blood pressure of 148/105 mmHg, clear consciousness, a 15-cm long surgical scar, a 6 cm × 3 cm cystic mass with fluctuation feeling on the back, backache, and percussion pain in the back, the disappearance of cremasteric reflex, bilateral positive pyramidal tract sign, and left and right lower extremity muscle strength of Grade 3 and Grade 2, respectively. Preoperative brain magnetic resonance imaging (Fig. 1A) and computed tomography (CT, Fig. 1B) showed no obvious abnormalities. The preoperative thoracic vertebra magnetic resonance imaging (Fig. 1C) revealed the following: 1. roundish nodules in the right foramen intervertebral of T 4-5, which were recurrent or residual tumors, and 2. subcutaneous hydrops in the dorsal side of the T3-8 vertebrae. The patient underwent thoracic vertebrae tumor excision, vertebral canal decompression, and injection of bone cement. The course of the anesthesia and surgery was smooth. The weakness of both lower limbs improved after surgery, and he did not report any other discomfort. Postoperative pathology suggested thoracic vertebral metastasis of hepatocellular carcinoma. We extracted 150 mL of bloody fluid from the cystic mass on his back on postoperative day 10, and 120 mL of bloody fluid was extracted again three days later; 100 mL of bloody fluid was extracted again after another interval of three days. He subsequently had a severe headache and vomiting that afternoon. The attending doctor immediately consulted a neurologist, who considered the possibility of intracranial hypertension, and suggested performing a head CT and discontinuing puncture aspiration. An emergency brain CT scan revealed a massive pneumocephalus (Figs. 1D and E). The patient was administered 128 mL intravenous mannitol every eight hours for five days; he was placed on bed rest and provided rehydration. His headache was relieved, and a head CT reexamination (Fig. 1 F) after five days of treatment showed that the pneumocephalus disappeared. He was then discharged. The patient was satisfied with the diagnosis and treatment.

## 4 Discussion

Pneumocephalus is an unusual postoperative complication of spinal surgery and is a potential cause of headache in the postoperative period. Sudden frontal headache is the dramatic initial symptom of pneumocephalus, as seen in our case. We speculate that repeated drainage with percutaneous aspiration after spinal surgery allowed ingress of air into the subarachnoid space via a hidden dural defect. The possibility of pneumocephalus cannot be excluded postoperatively, even 16 days later. The early diagnosis of pneumocephalus is crucial to avoid a severe consequence.

### 4.1 Main symptoms

The incidence of spinal surgery-related pneumocephalus is unknown because a cranial CT after spine surgery is not routinely performed. Sudden headache,^[4,5,7,8]^ especially a severe frontal headache,^[9]^ is the most frequent pneumocephalus presentation, as seen in our case. The headache is often accompanied by nausea, vomiting, and even coma^[10]^ or brain death.^[11]^ The specific symptoms are related to the volume and location of pneumocephalus. The occurrence of pneumocephalus 16 days after surgery observed in our case has not been reported previously.

### 4.2 Causes of pneumocephalus

Pneumocephalus of spinal origin may be associated with fractures caused by spinal trauma,^[12]^ operative injury^[3,4,7,8,10]^Table 1, tumors, application of H_2_O_2_,^[1,2]^ increased intra-abdominal pressure,^[6]^ or some diagnostic and therapeutic procedures, such as a lumbar puncture. Iatrogenic pneumocephalus^[1–4,7,8,10,11]^ are not rare during spinal operation. Postoperative pneumocephalus is often caused by dural injury.^[3,4]^ The presence of a dural injury was probably overlooked in our case.

**Table 1:** Clinical characteristics of the cases of pneumocephalus occurring during spinal surgery.

	Age, years/sex	Type of surgery	Initial symptom	Existence of dural tear/ injury	Cause of pneumocephalus	Brain CT findings	Main treatment	Outcomes
Zou et al.^[1]^	40/female	Lumbar interbody fusion surgery	Drowsiness, dilated pupils, and limb paralysis after operation.	No dural tearing was observed	Incision was soaked with H_2_O_2_ solution	Intracranial air trapped in the right frontal lobe and multiple cerebral infarction foci	–	Death
Kleffmann^[2]^	81/female	Lumbar spinal stenosis surgery	Somnolent and severe tetraparesis	Dural tear	Application of H_2_O_2_	Subdural air entrapment in the posterior cranial fossa and supratentorial and frontal regions	–	Death
Gader et al.^[3]^	40/female	Discal herniation's surgery	Autonomic dysfunction at postoperative 6 hours, followed by agitation, and seizures	Operation caused CSF leakage.	The dura mater was accidentally injured	Presence of frontal interhemispheric rounded hypodensities	–	Pneumocephalus disappeared in repeated CT on postoperative day 1.
Özdemir ^[4]^	25/male	Resection of lumbar vertebra tumor	Severe headache and vomiting on postoperative day 3	Operation caused dural injury	Possible dural injury	Numerous air density was shown in the convexity area	Definite bed rest and plenty of fluid replacement	Rapid recovery of symptoms after 3 d
Ayberk et al. ^[5]^	55/female	Spinal fusion	Headache and nausea on postoperative day 2	No dural defects were found	Increased intra-abdominal pressure	“Mount Fuji” sign	Oxygen therapy, hydration, bed rest, and analgesics.	–
Gauthé et al.^[6]^	69/male	Lumbar decompression surgery	A generalized seizure on postoperative day 1	No CSF leakage during surgery.	Drain	A voluminous pneumorachis	Anti-epileptic therapy, bed rest, hydration, oxygen therapy	Discharged without neurological deficit after 10 d
Yun et al. ^[7]^	59/male	Lumbar surgery for spinal stenosis	Headache and dizziness on postoperative day 2.	CSF leak following dural tear.	May be related with misplacement of a screw	Significant air in the frontal region, several cisterns, and intraventricle areas	Medication and hydration.	Symptoms were resolved within 2 wk
Son et al. ^[8]^	45/female	Thoracic spine tumor's surgery	High fever, headache, and suspicious neck stiffness	No CSF leakage was observed	A metallic device previously placed.	Severe pneumocephalus in subarachnoid spaces and ventricles	–	–
Ozturk et al. ^[10]^	23/female	Thoracolumbar scoliosis's surgery	Consciousness level deteriorated and unresponsive at postoperative 6 hours.	Inadvertent dural injury	Misplacement of a screw	Massive pneumocephalus in subarachnoid spaces, basal cisterns, and the ventricular system.	Dural tear repair	No neurological deficits after 4 wk

CSF = cerebrospinal fluid, CT = computed tomography, H_2_O_2_ = hydrogen peroxide; –, not mentioned. “Mount Fuji” sign is image characterization of tension pneumocephalus.

### 4.3 Physiopathology

The surgery or tumor may have caused a hidden dural injury in our patient, and repeated percutaneous aspiration probably caused local negative pressure, which resulted in CSF leakage from the dural injury. In this case, the cystic fluid did not decrease significantly after repeated percutaneous aspiration, which hints at CSF leakage. Pneumocephalus can develop through two mechanisms. Air enters the cranial cavity from dura leakage through the “inverted bottle” mechanism,^[13]^ where drainage of CSF leads to a negative intracranial pressure being replaced by the influx of air. Another mechanism is the ‘ball-valve” mechanism, where the air is forced through a dural defect but does not escape. A ball-valve mechanism may allow air to enter but not exit the cranial cavity when the patient is in an upright position. These two mechanisms may exist simultaneously in patients.

### 4.4 Treatment

Generally, pneumocephalus of small volume or some tension pneumocephalus without severe symptoms can be treated conservatively,^[4–7]^Table 1. Conservative treatment include bed rest, avoiding the Valsalva maneuver (i.e., coughing and sneezing), abundant fluid replacement, and administering analgesics and osmotic diuretics such as mannitol. Oxygen therapy facilitates denitrogenation and reabsorption of pneumocephalus.^[6]^ Hyperbaric oxygen therapy (set at 2.5 atmospheres, 110 minutes) may be a better choice to facilitate reabsorption of pneumocephalus.^[14]^ Pneumocephalus usually resolves after several days^[3,4]^ to several weeks.^[7,10]^ When clinical symptoms such as intracranial hypertension and unconsciousness indicate a threat to life, urgent decompression should be considered to relieve intracranial hypertension. Dural tears require a meticulous intraoperative repair.^[10,15]^

### 4.5 Prevention

To prevent pneumocephalus during spinal surgery, we suggest that the head be kept in a head-down position, lower than that of the operative field. The dural sac should be soaked with irrigation saline. It is important to maintain good suction power during the suturing of the dura. A watertight dural closure using various tissue adhesives can minimize the risk of pneumocephalus.

Based on a PubMed search for “pneumocephalus” and “spine,” ours could be the first study to report on such a case. One shortcoming is that we did not perform repeated thoracic vertebra CTs when the pneumocephalus occurred. With this information, we might have found intraspinal pneumatosis.

In conclusion, dural injury during spinal surgery should be included in a list of potential causes of pneumocephalus. Wound exudates and cystic fluid after spinal surgery should be differentiated from CSF leakage, and reckless percutaneous aspirations should be avoided. Drainage with percutaneous aspiration after spinal surgery favors the formation of pneumocephalus through a hidden dural defect. The possibility of pneumocephalus cannot be entirely excluded 16 days after the operation. Early diagnosis of pneumocephalus is crucial to avoid severe consequences.

## Author contributions

**Conceptualization:** Liming Cao.

**Formal analysis:** Qi Sheng.

**Funding acquisition:** Yu Zhao, Xibao Tong.

**Resources:** Yu Zhao, Liming Cao.

**Supervision:** Ruifang Liu.

**Visualization:** Gaolei Dong.

**Writing – review & editing:** Liming Cao.
